# Global trends and forecasts of nonalcoholic steatohepatitis causing liver cancer incidence and deaths

**DOI:** 10.3389/fonc.2025.1623789

**Published:** 2026-01-19

**Authors:** Tao Cui, Chenyue Guan, Kun Song, Jiangtao Yu

**Affiliations:** 1 Department of Hepatobiliary and Pancreatic Surgery, Fuyang People’s Hospital, Fuyang, Anhui, China; 2 Department of Hepatobiliary and Pancreatic Surgery, the Affiliated Fuyang People's Hospital of Wannan Medical College, Fuyang, Anhui, China

**Keywords:** MASLD, hepatocellular carcinoma, disability-adjusted life years, socio-demographic index, GBD

## Abstract

**Background:**

Metabolic dysfunction-associated steatotic liver disease (MASLD), particularly its progressive form, nonalcoholic steatohepatitis (NASH), has emerged as a leading cause of hepatocellular carcinoma (HCC). The burden of MASLD is increasing rapidly in parallel with the rising global prevalence of obesity and metabolic syndrome, affecting approximately 30% of the world’s population.

**Objective:**

This study aimed to quantify global trends in NASH-related liver cancer incidence, mortality, and disability-adjusted life years (DALYs) from 1990 to 2021 and to project the future disease burden through 2035.

**Methods:**

Data from the Global Burden of Disease Study 2021, encompassing 204 countries, were utilized to evaluate age-standardized incidence (ASIR), mortality (ASMR), and DALY rates (ASDR) across 21 regions and five Socio-demographic Index (SDI) categories, with stratification by age, sex, and geographical location. Future trends were projected using statistical modeling approaches, while decomposition analysis was employed to identify the key drivers of changes in disease burden.

**Results:**

From 1990 to 2021, global ASIR of NASH-related HCC increased by 25% (0.4-0.5 per 100,000), with an EAPC of +3.5% (95% UI: 3.4-3.6). ASMR rose by 150% (0.2-0.5 per 100,000; EAPC + 3.2%), and ASDR increased by 20% (9.6-11.5 per 100,000; EAPC + 2.9%).Regional disparities: Australasia saw the steepest rise (ASIR + 200%, EAPC + 7.2%; ASMR + 150%, EAPC + 6.9%). High-income North America followed (ASIR + 160%, EAPC + 5.3%).Age/sex differences: Incidence peaked in ages 85–89 (6.31 per 100,000 men; 5.56 per 100,000 women). Men had higher burden, for example, 55–59 years: male ASIR 1.28 vs. female 0.87 per 100,000. but gender gaps narrowed after age 75.Risk drivers: Metabolic factors such as high fasting glucose accounted for 29.7% of deaths in ages 80–84; smoking contributed 11.1% in ages 55–59.Projections: By 2035, ASIR will rise to 0.74 per 100,000 (+7% from 2021), driven by aging, population growth, and epidemiological changes (52% of incidence increase globally).

**Conclusion:**

NASH-related HCC burden exhibits accelerating global growth, concentrated in high-SDI regions. Prioritizing metabolic management and early screening is critical to mitigate future burden.

## 1 Introduction

Non-alcoholic steatohepatitis, the progressive form of metabolic dysfunction-associated steatotic liver disease, is a chronic liver condition characterized by hepatic fat accumulation, inflammation, hepatocyte injury, and variable degrees of fibrosis (1–3). Against the backdrop of a rising global prevalence of obesity, metabolic syndrome, and type 2 diabetes—affecting over 30% of the world’s population—MASLD has become the most common chronic liver disease worldwide (4–6). Epidemiological evidence confirms NASH as a critical etiological factor for HCC (7, 8). Recent studies indicate that NASH-related HCC incidence has increased by 2.3-fold over the past 15 years, now accounting for 10–20% of HCC cases in Western countries and 5–10% in Asia (9, 10). The progression from NASH to HCC involves multiple pathological mechanisms, primarily including metabolic dysregulation, chronic inflammation, and fibrogenesis (11). According to a recent meta-analysis, the global prevalence of MASLD is estimated at 30.2% (95% CI: 28.5–32.0) (4). This metabolic epidemic continues to drive the growing burden of NASH-related HCC, with studies reporting that 60–70% of NASH-HCC cases are associated with concurrent type 2 diabetes (12). The prognosis for HCC remains poor, with a five-year survival rate below 20%, underscoring challenges in both timely diagnosis and effective treatment (13). As a result, NASH-related HCC represents a substantial and growing public health and economic challenge, calling for urgent intervention strategies (14, 15).

## 2 Methodology

### 2.1 Data sources

Data on the global burden of liver cancer were obtained from the Global Burden of Disease Study 2021 (GBD 2021), which provides comprehensive estimates across 204 countries and territories (16, 17). NASH-related HCC cases were identified using ICD-10 codes (K74.6, C22.0) and defined by the following criteria: histopathological confirmation of NASH (NAS score ≥5), absence of other liver disease etiologies (viral hepatitis, alcoholic liver disease),GBD’s Cause of Death Ensemble modeling (CODEm) algorithm integrated vital registration, cancer registry, and hospital data to estimate cause-specific mortality (18). All data were extracted using the GBD Results Tool (https://ghdx.healthdata.org/gbd-results-tool) and the GBD Compare (https://vizhub.healthdata.org/gbd-compare/) (19) data visualization platform.

### 2.2 Burden estimation framework

The primary outcomes were age-standardized incidence rate (ASIR), age-standardized mortality rate (ASMR), and age-standardized disability-adjusted life year rate (ASDR), all expressed per 100,000 population and calculated using the GBD world standard population structure. The ASIR was estimated using DisMod-MR 3.0, a hierarchical Bayesian meta-regression tool designed to ensure consistency between incidence, prevalence, remission, and mortality for each disease (20). The ASMR was derived from the CODEm framework, with 95% uncertainty intervals (UI) reported (21). The ASDR, representing the total burden of disease, was calculated as the sum of years of life lost (YLL) and years lived with disability (YLD) (22).

### 2.3 Statistical analyses

#### 2.3.1 Statistical analysis trend analysis and stratification

Temporal trends in NASH-related HCC burden were analyzed from 1990 to 2021. Trends were stratified across five SDI categories(low, low-middle, middle, high-middle, high), 21 GBD geographical regions, and by age group and sex. The association between SDI and ASDR was assessed using Spearman’s rank correlation coefficient due to the non-normal distribution of the data (23).

#### 2.3.2 Projection modeling

Future trends in ASIR, ASMR, and ASDR from 2022 to 2035 were projected using a Bayesian Age-Period-Cohort model. The model structure was defined as log(λ_age,period_)=f(age)+g(period)+h(cohort), here cohort is defined as period minus age. Non-informative priors were selected for the age, period, and cohort effects, modeled as a random walk of order 2. Model convergence was confirmed using the Gelman-Rubin statistic, with a threshold of R^<1.05 across three Markov chains. The model’s predictive performance was validated through back-testing on data from 2016–2020, which yielded a mean absolute error = 0.08 per 100,000.

#### 2.3.3 Decomposition analysis

To quantify the drivers of changes in disease burden between 1990 and 2021, a decomposition analysis was performed following Das Gupta’s method. This analysis attributed changes to three components: population growth, population aging, and epidemiological changes. The contribution of each driver was calculated at both the global and SDI-regional levels. The formula for population growth was ΔB_pop_=(P_2021_−P_1990_)×R_1990,_ The formula for Aging was ΔB_age_=∑[(R_a,2021_−R_a,1990_)×P_a,1990_] and The formula for Epidemiological change was ΔB_epi_=∑[(R_a,2021_−R_a,1990_)×(P_a,2021_−P_a,1990_)].

where P=population, R=age-specific rate, a= age group.

#### 2.3.4 Risk attribution

The contribution of specific risk factors to NASH-related HCC burden was assessed using the GBD’s comparative risk assessment (CRA) framework. This method calculated the population attributable fractions (PAF) for metabolic risks, including high fasting plasma glucose and high body-mass index, as well as behavioral risks such as smoking (24–26).

## 3 Results

### 3.1 Overall trends in the global burden of disease in the development of hepatocellular carcinoma attributable to MASLD

To assess the global GBD and trends in liver cancer due to MASLD, incident for rates, mortality rates, and DALYs liver cancer due to MASLD were calculated 1990 and for 2021, and EAPC were used to show time trends from 1990 to 2021.

**Figure 1:**
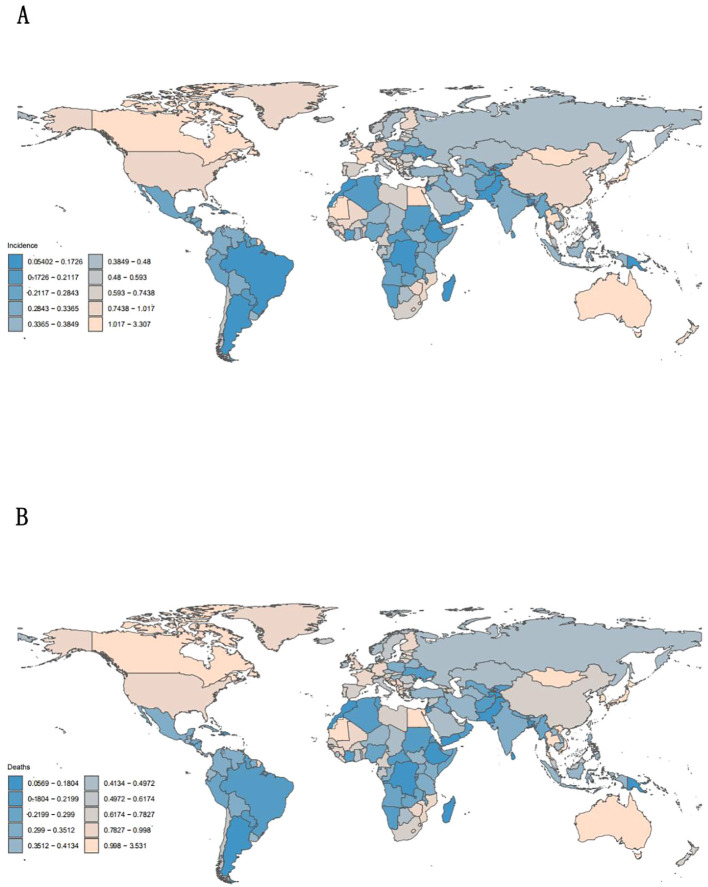
Incidence and mortality of diseases worldwide and by region, 2021.

Global burden accelerated substantially, with age-standardized incidence (ASIR) rising by 25% (0.4-0.5 per 100,000; EAPC + 3.5%) and mortality (ASMR) increasing 150% (0.2-0.5 per 100,000) from 1990 to 2021. As shown in **Figure 1**, Globally, the incidence, mortality and DALYs of liver cancer caused by nonalcoholic fatty liver disease are on the rise, with the global incidence (**Table 1**) increasing from 0.4 (95% UI: 0.3-0.5) to 0.5 (95% UI: 0.3-0.5) between 1990 and 2021, with an average annual percentage change of 3.5% (95% UI: 3.5-3.6). Among them, the incidence rate increased from 0.4 (95% UI: 0.3-0.5) to 0.5 (95% UI: 0.4-0.7) in men and from 0.3 (95% UI: 0.3-0.4) to 0.4 (95% UI: 0.4-0.5) in women, with persistent gender differences. The global average annual mortality increase from 1990 to 2021 was 3.2% (95% CI: 3.1-3.3), with the highest EAPC in Australasia (6.9; 95% CI: 6.8-7.0), the mortality rate increased from 0.2 (95% CI: 0.1-0.2) in 1990 to 0.5 (95% CI: 0.4-0.7) in 2021, an increase of 150%; High Income North America (EAPC: 5.1; 95% CI: 5.1-5.2) showed similarly significant increases in mortality, with combined male and female mortality rates in 2021 (0.5; 95% CI: 0.4-0.6) compared with 1990 (0.2; 95% CI:0.2-0.2) increased by 150%. Overall global DALYs increased from 9.6 (95% UI:7.7-11.9) in 1990 to 11.5 (95% UI:9.4-13.8) in 2021, with an average annual percentage change of 2.9% (95% CI: 2.8-3.0), total DALYs for men and women in 2021 (11.0 per 100,000; 95%UI: 9.3-12.9) compared with 1990 (4.6 per 100,000; UI:4.0-5.3) an increase of 139%. DALYs were consistently higher in males than in females, but EAPC was similar in both sexes (2.9 vs. 2.8) for women.

**Table 1:** Disease incidence, mortality, DALYs and EAPC in 21 regions and 5 SDI worldwide.

incidence									
location_name	metric_name	1990-Male	1990-Female	1990-Both	2021-Male	2021-Female	2021-Both	EAPC	EAPCs
Global	Rate	0.4(0.3,0.5)	0.3(0.3,0.4)	0.4(0.3,0.4)	0.5(0.4,0.7)	0.4(0.4,0.5)	0.5(0.4,0.6)	3.547232217	3.5(3.5,3.6)
East Asia	Rate	0.5(0.4,0.6)	0.4(0.3,0.6)	0.5(0.4,0.6)	0.6(0.5,0.8)	0.5(0.4,0.6)	0.5(0.4,0.7)	3.695994092	3.7(3.5,3.9)
Southeast Asia	Rate	0.6(0.4,0.9)	0.5(0.4,0.7)	0.6(0.4,0.8)	0.8(0.5,1.1)	0.5(0.3,0.8)	0.6(0.5,0.9)	3.457863211	3.5(3.4,3.5)
Oceania	Rate	0.4(0.2,0.8)	0.4(0.2,0.8)	0.4(0.2,0.7)	0.3(0.2,0.7)	0.3(0.2,0.6)	0.3(0.2,0.6)	2.591261196	2.6(2.5,2.7)
Central Asia	Rate	0.5(0.4,0.8)	0.5(0.3,0.6)	0.5(0.3,0.7)	0.5(0.4,0.8)	0.6(0.4,0.8)	0.6(0.4,0.8)	1.985596732	2.0(1.8,2.1)
Central Europe	Rate	0.2(0.2,0.3)	0.3(0.2,0.4)	0.2(0.2,0.3)	0.3(0.2,0.4)	0.3(0.2,0.3)	0.3(0.2,0.4)	1.761247659	1.8(1.6,1.9)
Eastern Europe	Rate	0.2(0.1,0.2)	0.1(0.1,0.1)	0.1(0.1,0.2)	0.3(0.2,0.3)	0.2(0.1,0.2)	0.2(0.2,0.2)	2.35246895	2.4(2.1,2.6)
High-income Asia Pacific	Rate	0.9(0.8,1.2)	0.6(0.5,0.8)	0.8(0.6,1.0)	0.7(0.6,1.0)	0.5(0.4,0.7)	0.6(0.5,0.8)	1.676288143	1.7(1.3,2.1)
Australasia	Rate	0.2(0.1,0.3)	0.1(0.1,0.2)	0.2(0.1,0.2)	0.7(0.5,1.0)	0.5(0.4,0.7)	0.6(0.4,0.8)	7.236094498	7.2(7.0,7.4)
Western Europe	Rate	0.2(0.2,0.3)	0.2(0.1,0.2)	0.2(0.2,0.3)	0.4(0.3,0.6)	0.3(0.2,0.4)	0.4(0.3,0.5)	3.605115288	3.6(3.5,3.7)
Southern Latin America	Rate	0.1(0.0,0.1)	0.1(0.0,0.1)	0.1(0.0,0.1)	0.2(0.1,0.3)	0.2(0.1,0.2)	0.2(0.1,0.3)	6.147050655	6.1(5.9,6.3)
High-income North America	Rate	0.3(0.2,0.3)	0.2(0.2,0.2)	0.2(0.2,0.3)	0.7(0.6,0.9)	0.5(0.4,0.5)	0.6(0.5,0.7)	5.29273983	5.3(5.2,5.4)
Caribbean	Rate	0.1(0.1,0.2)	0.2(0.2,0.3)	0.2(0.1,0.3)	0.2(0.1,0.3)	0.2(0.1,0.3)	0.2(0.1,0.3)	2.328888457	2.3(2.1,2.6)
Andean Latin America	Rate	0.1(0.1,0.2)	0.4(0.3,0.5)	0.3(0.2,0.4)	0.2(0.1,0.3)	0.5(0.3,0.7)	0.3(0.2,0.5)	4.267233796	4.3(4.0,4.5)
Central Latin America	Rate	0.2(0.1,0.2)	0.3(0.2,0.4)	0.2(0.2,0.3)	0.3(0.2,0.3)	0.3(0.2,0.4)	0.3(0.2,0.4)	4.728182696	4.7(4.5,5.0)
Tropical Latin America	Rate	0.1(0.1,0.1)	0.1(0.1,0.2)	0.1(0.1,0.1)	0.2(0.1,0.2)	0.2(0.1,0.2)	0.2(0.1,0.2)	4.98642965	5.0(4.7,5.3)
North Africa and Middle East	Rate	0.3(0.2,0.5)	0.5(0.3,0.8)	0.4(0.2,0.7)	0.7(0.5,1.0)	0.6(0.4,0.8)	0.6(0.4,0.9)	4.840458559	4.8(4.6,5.1)
South Asia	Rate	0.2(0.2,0.3)	0.2(0.2,0.3)	0.2(0.2,0.3)	0.4(0.3,0.5)	0.3(0.3,0.4)	0.4(0.3,0.4)	4.596924714	4.6(4.5,4.7)
Central Sub-Saharan Africa	Rate	0.5(0.2,1.1)	0.5(0.2,1.3)	0.5(0.2,1.1)	0.4(0.1,0.9)	0.4(0.2,1.1)	0.4(0.2,1.0)	1.89389314	1.9(1.6,2.1)
Eastern Sub-Saharan Africa	Rate	0.5(0.3,0.8)	0.9(0.6,1.2)	0.7(0.5,1.0)	0.5(0.3,0.9)	0.9(0.6,1.2)	0.7(0.5,1.0)	2.527867584	2.5(2.4,2.6)
Southern Sub-Saharan Africa	Rate	0.6(0.2,1.1)	0.6(0.4,0.9)	0.6(0.4,0.9)	1.3(1.0,1.7)	0.9(0.7,1.1)	1.0(0.8,1.3)	3.557679775	3.6(2.9,4.2)
Western Sub-Saharan Africa	Rate	1.0(0.5,1.5)	1.3(0.7,2.4)	1.2(0.7,2.0)	0.9(0.6,1.2)	1.3(1.0,1.7)	1.1(0.8,1.5)	2.135669472	2.1(2.0,2.3)
High-middle SDI	Rate	0.4(0.3,0.4)	0.3(0.2,0.3)	0.3(0.2,0.4)	0.5(0.4,0.6)	0.3(0.3,0.4)	0.4(0.3,0.5)	3.131560684	3.1(3.0,3.3)
High SDI	Rate	0.4(0.3,0.5)	0.3(0.2,0.3)	0.3(0.3,0.4)	0.6(0.5,0.8)	0.4(0.3,0.5)	0.5(0.4,0.6)	3.618234557	3.6(3.4,3.8)
Low-middle SDI	Rate	0.3(0.2,0.4)	0.4(0.3,0.5)	0.3(0.2,0.5)	0.5(0.3,0.6)	0.5(0.4,0.6)	0.5(0.4,0.6)	3.905523016	3.9(3.8,4.0)
Low SDI	Rate	0.5(0.3,0.8)	0.7(0.4,1.2)	0.6(0.4,1.0)	0.5(0.3,0.7)	0.7(0.5,1.0)	0.6(0.4,0.8)	2.37309237	2.4(2.3,2.5)
Middle SDI	Rate	0.4(0.3,0.5)	0.4(0.3,0.5)	0.4(0.3,0.5)	0.6(0.4,0.7)	0.5(0.4,0.6)	0.5(0.4,0.6)	3.899264303	3.9(3.7,4.1)

death									
location_name	metric_name	1990-Male	1990-Female	1990-Both	2021-Male	2021-Female	2021-Both	EAPC	EAPCs
Global	Rate	0.4(0.3,0.5)	0.4(0.3,0.5)	0.4(0.3,0.5)	0.5(0.4,0.6)	0.4(0.4,0.5)	0.5(0.4,0.6)	3.193795007	3.2(3.1,3.3)
East Asia	Rate	0.5(0.4,0.7)	0.5(0.4,0.6)	0.5(0.4,0.6)	0.6(0.4,0.8)	0.5(0.3,0.6)	0.5(0.4,0.6)	2.804396726	2.8(2.6,3.0)
Southeast Asia	Rate	0.7(0.5,0.9)	0.5(0.4,0.7)	0.6(0.4,0.8)	0.8(0.6,1.2)	0.6(0.4,0.8)	0.7(0.5,0.9)	3.35946898	3.4(3.3,3.4)
Oceania	Rate	0.4(0.2,0.9)	0.4(0.2,0.8)	0.4(0.2,0.8)	0.4(0.2,0.7)	0.4(0.2,0.6)	0.4(0.2,0.6)	2.699332418	2.7(2.6,2.8)
Central Asia	Rate	0.6(0.4,0.8)	0.5(0.3,0.7)	0.5(0.4,0.8)	0.6(0.4,0.9)	0.6(0.4,0.9)	0.6(0.4,0.9)	1.957662295	2.0(1.9,2.1)
Central Europe	Rate	0.2(0.2,0.3)	0.3(0.2,0.4)	0.3(0.2,0.4)	0.3(0.2,0.4)	0.3(0.2,0.4)	0.3(0.2,0.4)	1.583239309	1.6(1.4,1.7)
Eastern Europe	Rate	0.2(0.1,0.2)	0.1(0.1,0.1)	0.1(0.1,0.2)	0.3(0.2,0.3)	0.2(0.2,0.2)	0.2(0.2,0.3)	2.497766626	2.5(2.3,2.7)
High-income Asia Pacific	Rate	0.8(0.7,1.1)	0.6(0.5,0.8)	0.7(0.6,0.9)	0.6(0.4,0.7)	0.4(0.3,0.5)	0.5(0.4,0.6)	2.209542334	2.2(1.9,2.5)
Australasia	Rate	0.2(0.1,0.3)	0.1(0.1,0.2)	0.2(0.1,0.2)	0.6(0.4,0.8)	0.5(0.3,0.7)	0.5(0.4,0.7)	6.902246055	6.9(6.8,7.0)
Western Europe	Rate	0.2(0.2,0.3)	0.2(0.1,0.2)	0.2(0.2,0.3)	0.4(0.3,0.5)	0.3(0.2,0.4)	0.3(0.2,0.4)	3.035669706	3.0(2.9,3.2)
Southern Latin America	Rate	0.1(0.0,0.1)	0.1(0.0,0.1)	0.1(0.0,0.1)	0.2(0.1,0.3)	0.2(0.1,0.2)	0.2(0.1,0.3)	5.253402063	5.3(4.9,5.6)
High-income North America	Rate	0.2(0.2,0.3)	0.2(0.1,0.2)	0.2(0.2,0.2)	0.6(0.5,0.7)	0.4(0.3,0.5)	0.5(0.4,0.6)	5.127194071	5.1(5.1,5.2)
Caribbean	Rate	0.2(0.1,0.2)	0.3(0.2,0.3)	0.2(0.1,0.3)	0.2(0.1,0.3)	0.2(0.2,0.3)	0.2(0.2,0.3)	2.505036044	2.5(2.3,2.7)
Andean Latin America	Rate	0.1(0.1,0.2)	0.4(0.3,0.6)	0.3(0.2,0.4)	0.2(0.1,0.3)	0.5(0.3,0.7)	0.4(0.2,0.5)	3.923072231	3.9(3.7,4.1)
Central Latin America	Rate	0.2(0.2,0.3)	0.3(0.2,0.4)	0.3(0.2,0.3)	0.3(0.2,0.4)	0.4(0.3,0.4)	0.3(0.3,0.4)	4.353960612	4.4(4.1,4.6)
Tropical Latin America	Rate	0.1(0.1,0.1)	0.2(0.1,0.2)	0.1(0.1,0.2)	0.2(0.1,0.2)	0.2(0.1,0.2)	0.2(0.1,0.2)	4.084459266	4.1(3.7,4.4)
North Africa and Middle East	Rate	0.4(0.2,0.6)	0.5(0.3,0.9)	0.4(0.3,0.7)	0.7(0.5,1.0)	0.7(0.5,0.9)	0.7(0.5,0.9)	4.202462892	4.2(4.0,4.4)
South Asia	Rate	0.3(0.2,0.3)	0.2(0.2,0.3)	0.2(0.2,0.3)	0.4(0.3,0.5)	0.4(0.3,0.4)	0.4(0.3,0.5)	4.305745959	4.3(4.2,4.4)
Central Sub-Saharan Africa	Rate	0.5(0.2,1.2)	0.6(0.2,1.4)	0.5(0.2,1.2)	0.4(0.2,1.0)	0.5(0.2,1.3)	0.5(0.2,1.1)	1.90412701	1.9(1.8,2.0)
Eastern Sub-Saharan Africa	Rate	0.5(0.3,0.9)	1.0(0.7,1.4)	0.8(0.5,1.1)	0.6(0.3,0.9)	1.0(0.7,1.3)	0.8(0.6,1.1)	2.687884853	2.7(2.6,2.8)
Southern Sub-Saharan Africa	Rate	0.6(0.3,1.2)	0.7(0.5,1.0)	0.7(0.4,1.0)	1.5(1.1,1.9)	1.0(0.8,1.2)	1.1(0.9,1.4)	4.252318574	4.3(3.8,4.7)
Western Sub-Saharan Africa	Rate	1.1(0.6,1.7)	1.5(0.8,2.7)	1.3(0.7,2.2)	1.0(0.7,1.3)	1.5(1.1,1.9)	1.2(0.9,1.7)	2.241133158	2.2(2.2,2.3)
High-middle SDI	Rate	0.4(0.3,0.5)	0.3(0.2,0.3)	0.3(0.3,0.4)	0.4(0.3,0.6)	0.3(0.3,0.4)	0.4(0.3,0.5)	2.756155409	2.8(2.6,2.9)
High SDI	Rate	0.4(0.3,0.4)	0.3(0.2,0.3)	0.3(0.2,0.4)	0.5(0.4,0.6)	0.4(0.3,0.5)	0.4(0.3,0.5)	3.55998877	3.6(3.4,3.7)
Low-middle SDI	Rate	0.3(0.2,0.4)	0.4(0.3,0.6)	0.4(0.3,0.5)	0.5(0.4,0.6)	0.5(0.4,0.6)	0.5(0.4,0.6)	3.576831428	3.6(3.5,3.7)
Low SDI	Rate	0.6(0.4,0.9)	0.8(0.5,1.3)	0.7(0.4,1.1)	0.5(0.4,0.8)	0.8(0.6,1.1)	0.7(0.5,0.9)	2.406212914	2.4(2.3,2.5)
Middle SDI	Rate	0.5(0.4,0.6)	0.5(0.4,0.6)	0.5(0.4,0.6)	0.6(0.5,0.7)	0.5(0.4,0.6)	0.5(0.4,0.6)	3.24713108	3.2(3.1,3.4)

DALYs									
location_name	metric_name	1990-Male	1990-Female	1990-Both	2021-Male	2021-Female	2021-Both	EAPC	EAPCs
Global	Rate	10.3(8.1,12.7)	9.0(7.2,11.4)	9.6(7.7,11.9)	12.6(9.9,15.7)	10.5(8.6,12.6)	11.5(9.4,13.8)	2.934604712	2.9(2.8,3.0)
East Asia	Rate	14.3(11.2,18.2)	12.4(9.5,15.6)	13.4(10.8,16.5)	14.3(10.3,19.2)	10.5(8.0,14.0)	12.4(9.6,15.6)	2.537552007	2.5(2.3,2.8)
Southeast Asia	Rate	17.2(12.0,23.9)	13.3(9.9,17.8)	15.2(11.0,20.6)	20.0(14.0,29.2)	13.1(8.1,18.2)	16.4(11.5,22.4)	3.121580663	3.1(3.0,3.2)
Oceania	Rate	9.7(5.3,21.5)	10.1(5.1,20.1)	9.9(5.4,19.8)	8.7(4.6,17.9)	9.0(5.4,15.1)	8.8(5.0,16.2)	2.531303781	2.5(2.4,2.6)
Central Asia	Rate	15.0(10.3,21.7)	13.0(9.0,17.7)	13.8(9.7,19.3)	14.3(9.5,20.9)	15.0(10.3,20.9)	14.7(10.1,21.0)	1.757668721	1.8(1.6,1.9)
Central Europe	Rate	5.7(4.0,8.1)	6.7(4.8,9.0)	6.3(4.5,8.5)	7.0(5.0,9.7)	6.0(4.3,7.8)	6.4(4.7,8.7)	1.357954324	1.4(1.1,1.6)
Eastern Europe	Rate	4.3(3.6,5.1)	3.2(2.7,3.7)	3.6(3.0,4.2)	6.8(5.7,8.1)	4.5(3.8,5.2)	5.4(4.6,6.3)	2.038104292	2.0(1.7,2.3)
High-income Asia Pacific	Rate	21.7(17.2,28.0)	13.6(10.4,17.4)	17.5(14.0,21.9)	12.0(9.2,16.3)	7.9(5.9,10.4)	9.9(7.7,12.9)	0.065775474	0.1(-0.4,0.5)
Australasia	Rate	4.4(3.2,6.0)	3.2(2.4,4.0)	3.7(2.8,4.9)	14.4(10.3,19.5)	11.1(8.1,14.7)	12.7(9.3,16.9)	6.515842596	6.5(6.4,6.6)
Western Europe	Rate	5.5(4.1,7.2)	3.8(3.0,4.8)	4.6(3.5,5.9)	8.1(6.0,11.0)	5.9(4.5,7.6)	6.9(5.3,9.1)	2.898219396	2.9(2.8,3.0)
Southern Latin America	Rate	1.7(1.1,2.5)	1.5(1.0,2.1)	1.6(1.1,2.2)	4.8(3.2,7.0)	3.9(2.7,5.2)	4.3(3.0,5.9)	5.872145396	5.9(5.6,6.1)
High-income North America	Rate	5.6(4.7,6.4)	3.9(3.4,4.4)	4.6(4.0,5.3)	13.4(11.3,15.9)	8.8(7.4,10.2)	11.0(9.3,12.9)	5.087146739	5.1(5.0,5.2)
Caribbean	Rate	3.7(2.5,5.1)	5.8(4.1,7.8)	4.8(3.4,6.5)	5.0(3.3,7.2)	5.3(3.6,7.2)	5.1(3.6,7.0)	2.230749008	2.2(1.9,2.5)
Andean Latin America	Rate	3.2(2.2,4.7)	9.6(6.7,13.2)	6.5(4.7,8.9)	4.3(2.8,6.2)	11.0(7.1,15.6)	7.8(5.1,11.2)	3.752278514	3.8(3.4,4.1)
Central Latin America	Rate	4.6(3.6,6.0)	7.4(5.7,9.4)	6.0(4.7,7.7)	6.9(5.4,8.5)	8.0(6.2,9.9)	7.5(5.9,9.2)	4.307280893	4.3(4.0,4.6)
Tropical Latin America	Rate	2.6(2.2,3.0)	3.7(3.2,4.2)	3.2(2.8,3.6)	3.9(3.3,4.5)	4.0(3.4,4.6)	4.0(3.4,4.6)	4.707147231	4.7(4.4,5.0)
North Africa and Middle East	Rate	9.1(6.2,14.4)	12.0(7.2,21.3)	10.6(6.7,17.5)	17.2(11.6,24.3)	15.7(11.4,20.9)	16.4(11.6,22.5)	4.606162923	4.6(4.4,4.8)
South Asia	Rate	6.6(5.2,8.1)	5.9(4.7,7.0)	6.3(5.1,7.5)	9.6(7.9,11.9)	9.1(7.5,10.7)	9.3(7.9,11.1)	4.210599483	4.2(4.1,4.3)
Central Sub-Saharan Africa	Rate	12.9(4.9,30.4)	14.3(5.6,33.9)	13.6(5.8,29.7)	9.9(3.8,23.5)	12.1(4.7,31.4)	11.1(4.6,25.6)	1.947005148	1.9(1.7,2.2)
Eastern Sub-Saharan Africa	Rate	13.6(8.4,21.9)	23.1(16.7,32.2)	18.4(12.9,25.7)	14.5(8.6,23.4)	22.7(16.1,30.3)	18.9(12.9,26.2)	2.525536908	2.5(2.4,2.6)
Southern Sub-Saharan Africa	Rate	15.8(6.5,29.2)	17.9(12.4,26.4)	16.9(10.4,25.5)	34.0(25.3,44.9)	23.5(18.3,30.1)	27.8(22.2,34.3)	3.357832011	3.4(2.6,4.1)
Western Sub-Saharan Africa	Rate	26.2(14.7,40.9)	34.9(19.2,63.3)	30.6(17.9,50.6)	23.1(16.1,32.2)	33.0(24.4,43.9)	28.3(20.9,37.6)	2.187754269	2.2(2.0,2.3)
High-middle SDI	Rate	9.9(7.9,12.1)	7.0(5.7,8.5)	8.4(6.9,10.0)	10.9(8.2,13.8)	7.5(6.0,9.4)	9.1(7.2,10.9)	2.298287852	2.3(2.2,2.4)
High SDI	Rate	8.8(7.0,11.2)	6.0(4.8,7.3)	7.3(5.9,9.1)	11.3(9.1,14.1)	8.1(6.6,9.9)	9.6(7.8,12.0)	2.832264529	2.8(2.7,3.0)
Low-middle SDI	Rate	8.4(6.3,11.3)	9.9(7.2,14.2)	9.2(6.8,12.7)	12.4(9.6,15.7)	12.7(10.2,15.6)	12.6(10.0,15.4)	3.672641074	3.7(3.6,3.8)
Low SDI	Rate	14.7(9.3,22.1)	19.1(12.3,30.8)	16.9(10.9,25.6)	13.2(8.9,19.1)	18.9(13.6,25.6)	16.1(11.5,22.3)	2.349517246	2.3(2.2,2.5)
Middle SDI	Rate	11.9(9.4,14.8)	11.4(9.1,13.9)	11.7(9.5,14.1)	14.1(10.8,18.0)	10.9(8.8,13.2)	12.5(9.9,15.2)	3.170297738	3.2(3.0,3.3)

During the period from 2015 to 2020, the growth of ASIR slowed significantly (EAPC + 0.2% per year, compared with +4.1% from 2010 to 2015), which might be attributed to: public health intervention measures and disruptions caused by COVID-19.Among the public health intervention measures are the 2016 EASL guidelines promoting the screening of MASLD in primary care and early detection driven by electronic health records in areas with high SDI. The interruption of COVID-19 led to a reduction in the volume of selective liver imaging and biopsy.

### 3.2 Regional trends in the burden of disease

**Figure 2:**
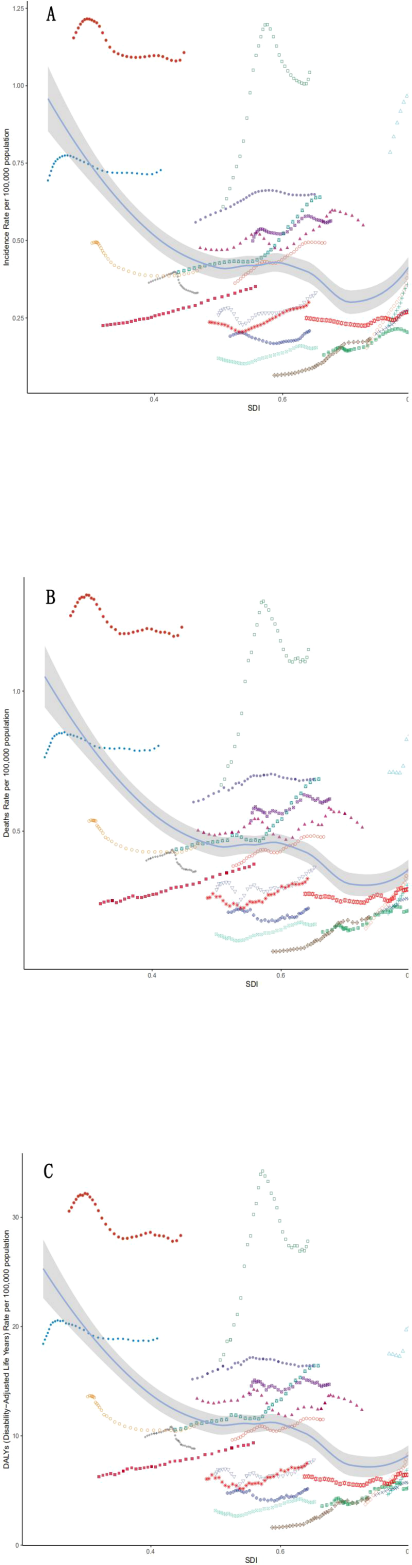
Incidence **(A)**, mortality **(B)** and DALYs **(C)** of the disease globally and in different SDI regions, 1990 to 2021.

Regional disparities defined disease distribution, as high-SDI regions exhibited extreme growth—Australasia led with 200% ASIR increase (EAPC + 7.2%) while sub-Saharan Africa (low-SDI) showed high mortality (0.8 per 100,000) despite low incidence (0.2 per 100,000). while temporal trends by age group appear in **Figure 2**, Australasia saw the fastest growth in ASIR (200%: 0.2-0.6 per 100,000;EAPC +7.2%), followed by high-income North America (ASIR + 160%; EAPC + 5.3%).The possible reasons for this difference mainly include: First, the high obesity rate: The adult obesity rate in Australia/New Zealand is 30%, while the global average is 13%.Secondly, enhance diagnostic capabilities: Since 2010, ultrasound/fiber scanning screening has been widely implemented. Additionally, for the elderly population: over 20% aged 65 and above accelerate the progression of NASH Sub-saharan Africa (low SDI) shows a completely different trend: low ASIR (0.2 per 100,000) but high ASMR (0.8 per 100,000), indicating delayed diagnosis and limited treatment accessibility (**Figure 1**).

**Figure 3:**
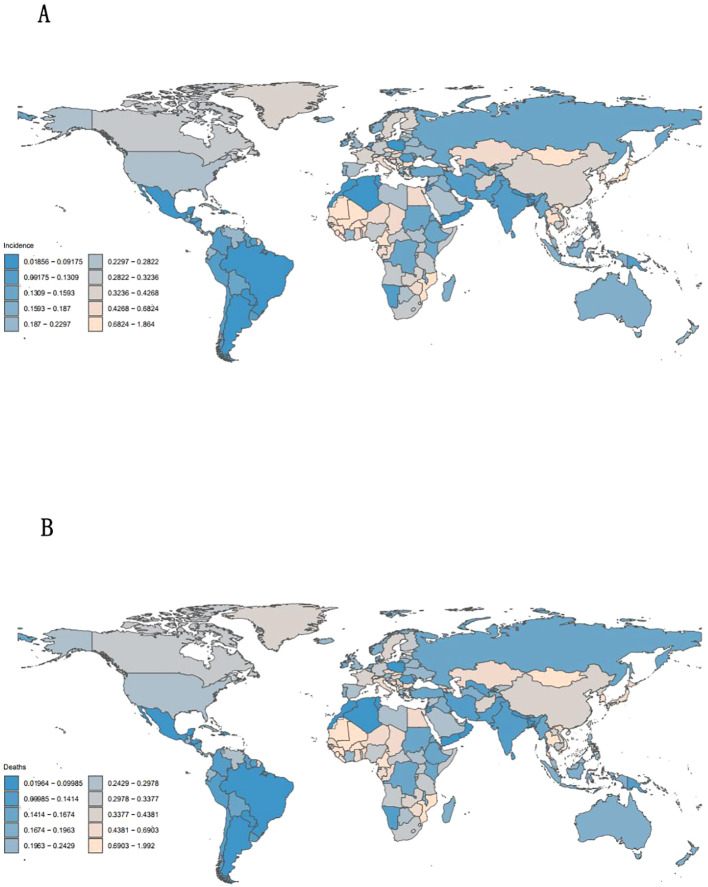
Incidence **(A)** and mortality **(B)** of diseases worldwide and by region, 2019.

The analysis of mortality rate (**Figure 2**) shows that the mortality rate in Australasia also ranks first in the world, surging from 0.2 (95%UI:0.1-0.2) in 1990 to 0.5 (95%UI:0.4-0.7) in 2021, with a relative increase of 150% and an EAPC of 6.9% (95%UI: 6.8-7.0), the growth rate of mortality in high-income North America was second only to Australasia. The mortality rate in high SDI areas increased significantly, the incidence rate in low SDI areas was relatively stable, and the growth rate was the fastest in low and medium SDI areas. Australasia and high-income North America lead in both incidence and mortality, while sub to Saharan Africa has less fluctuating incidence but high mortality, which may reflect delayed diagnosis or low treatment coverage.

Then we analyzed DALYs, among which Australasia had the highest EAPC (6.5; 95% CI: 6.4-6.6), its DALYs rate soared from 3.7 per 100,000 (95%UI:2.8-4.9) in 1990 to 12.7 per 100,000 (95%UI:9.3-16.9) in 2021, an increase of 243%. High Income North America (EAPC: 5.1; 95% CI: 5.0-5.2) the DALYs rate also increased significantly. Consistent with morbidity and mortality, DALYs in Australasia and high-income North America rank first in the world. The disease burden in high SDI areas was still heavy, with DALYs rising from 7.3 (95%UI:5.9-9.1) to 9.6 (95%UI:7.8-12.0) and EAPC 2.8% (95%UI:2.7-3.0). Low and medium SDI areas: DALYs increased the fastest, low SDI areas: DALYs is relatively stable.

### 3.3 Global trends by age and sex specificity

**Figure 4:**
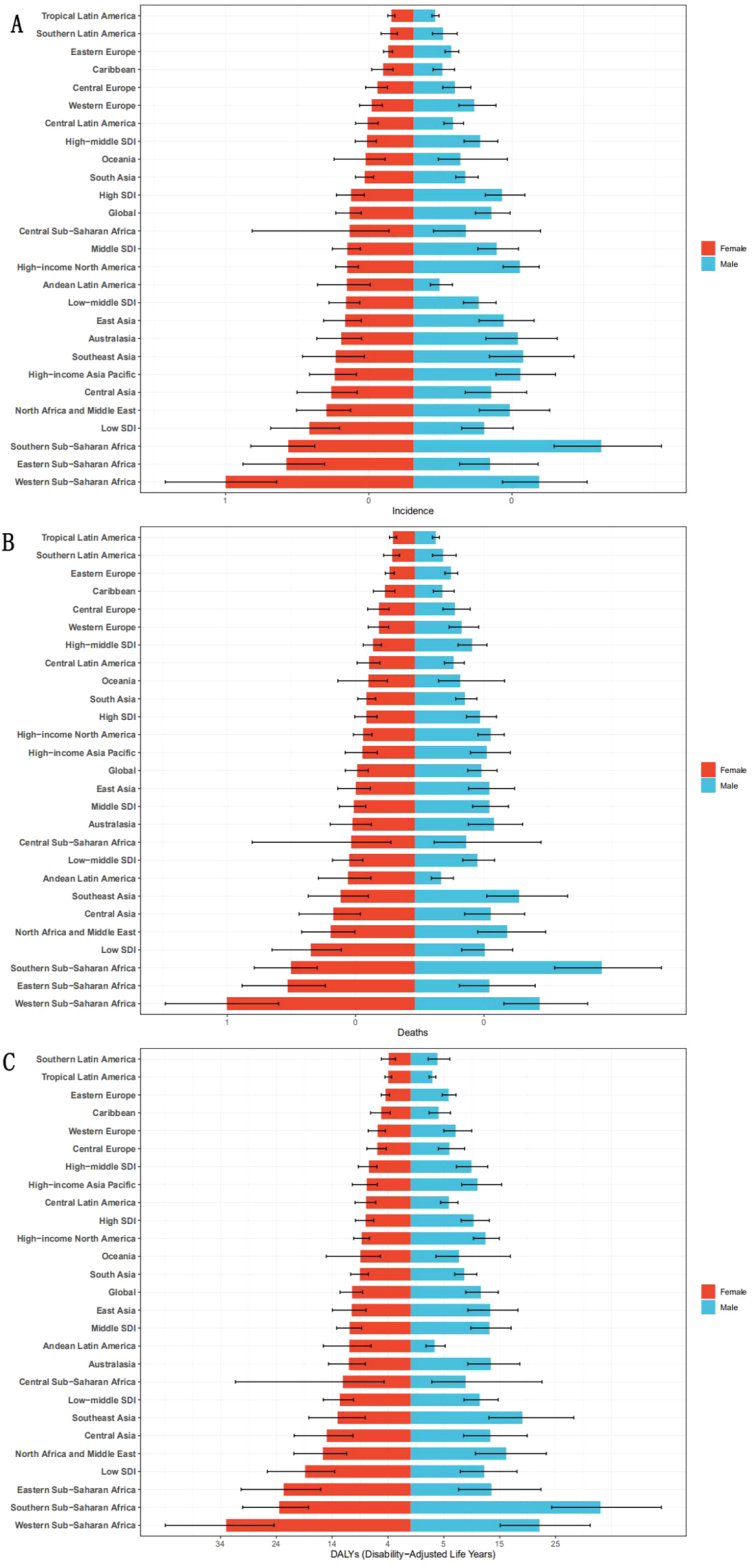
Incidence **(A)**, mortality **(B)** and DALYs **(C)** for males and females in all age groups for the disease in 2021.

Age and sex stratification revealed critical gradients, peaking at ages 85–89 years (6.31 per 100,000 men; 5.56 per 100,000 women) with narrowing gender gaps after age 75 (women ≥95 had 8.5% higher mortality than men).In terms of age distribution, the incidence showed a significant increase with age (**Figure 4**). From ages 15-19, cases begin to appear. Thereafter, the incidence rate continued to rise with age, reaching a peak in the 85–89 age group, with 6.31 per 100,000 men (95%UI:4.50-8.69) and 5.56 per 100,000 women (95%UI:3.69-7.56). In terms of gender, men have higher incidence than women in most age groups. Taking the 55–59 year old group as an example, the male incidence was 1.28 per 100,000 (95%UI:0.86-1.89), which was significantly higher than the female incidence of 0.87 per 100,000 (95%UI:0.61-1.19). The gender difference was reduced in the group over 75 years old, but men still dominated. In addition, the incidence was slightly higher in women aged 95+ (4.13 per 100,000) than in men (3.78 per 100,000), suggesting that The higher mortality in women ≥95 years (6.40 vs. 5.90 per 100,000 men) may relate to age-related comorbidities, though NASH-specific biological mechanisms require further validation. The peak number of cases was concentrated in the 65–69 age group, with 3363.6 cases in males (95%UI:2257.2-4581) and 3031.7 cases in females (95%UI:2137.6-4101.2).

The death rate also showed a stepwise increase with age. Male mortality peaked in the 90–94 year age group (7.46 per 100,000, 95%UI:4.95-10.78), followed closely by females in the same age group (6.48 per 100,000, 95%UI:4.07-9.35), and it is noteworthy that the mortality rate for females aged 95+ years (6.40 per 100,000, 6.40 per 100,000) was higher than that for females aged 95+ years. 95%UI:3.52- 10.17) was significantly higher than that of men (5.90 per 100,000, 95%UI:3.34-9.28), suggesting that the prognosis of older women may be worse. In terms of gender, men have higher mortality rates than women in most age groups. However, over the age of 75 years, the gender gap gradually Narrows, and there is even a higher mortality rate for women than for men. The peak number of deaths was concentrated in the 65–69 age group, with 3129 deaths in males (95%UI:2093.8-4268.3) and 2919.5 deaths in females (95%UI:2059.1-3937.4).

We then analyzed DALYs and found that the disease burden increased significantly with age, and there were significant gender differences. DALYs began to appear at the age of 15-19, but the value was relatively low. After that, DALYs increased rapidly with age. The peak of DALYs rate was 71.03% (95%UI:49.12-100.68) in the male group aged 80–84 years, and the number of DALYs cases was 26033.0 per 100,000. In females, the peak DALYs rate was 65.39% (95%UI:45.51-90.56) in the 80–84 age group, and the number of DALYs cases was 26032.9 per 100,000. In terms of gender differences, the number and rate of DALYs in males were higher than that in females in most age groups. However, at the age of 75 and above, the gender difference gradually Narrows, and there is even a phenomenon that the number of DALYs in women is higher than that in men. In addition, the DALYs rate of 90–94 years old group showed that the gap between 65.52% (95%UI: 43.44-94.58) in males and 56.78%(95%UI:35.55-81.87) in females was further narrowed, suggesting that the disease burden of elderly women increased significantly.

### 3.4 Global trends by regional time

**Figure 5:**
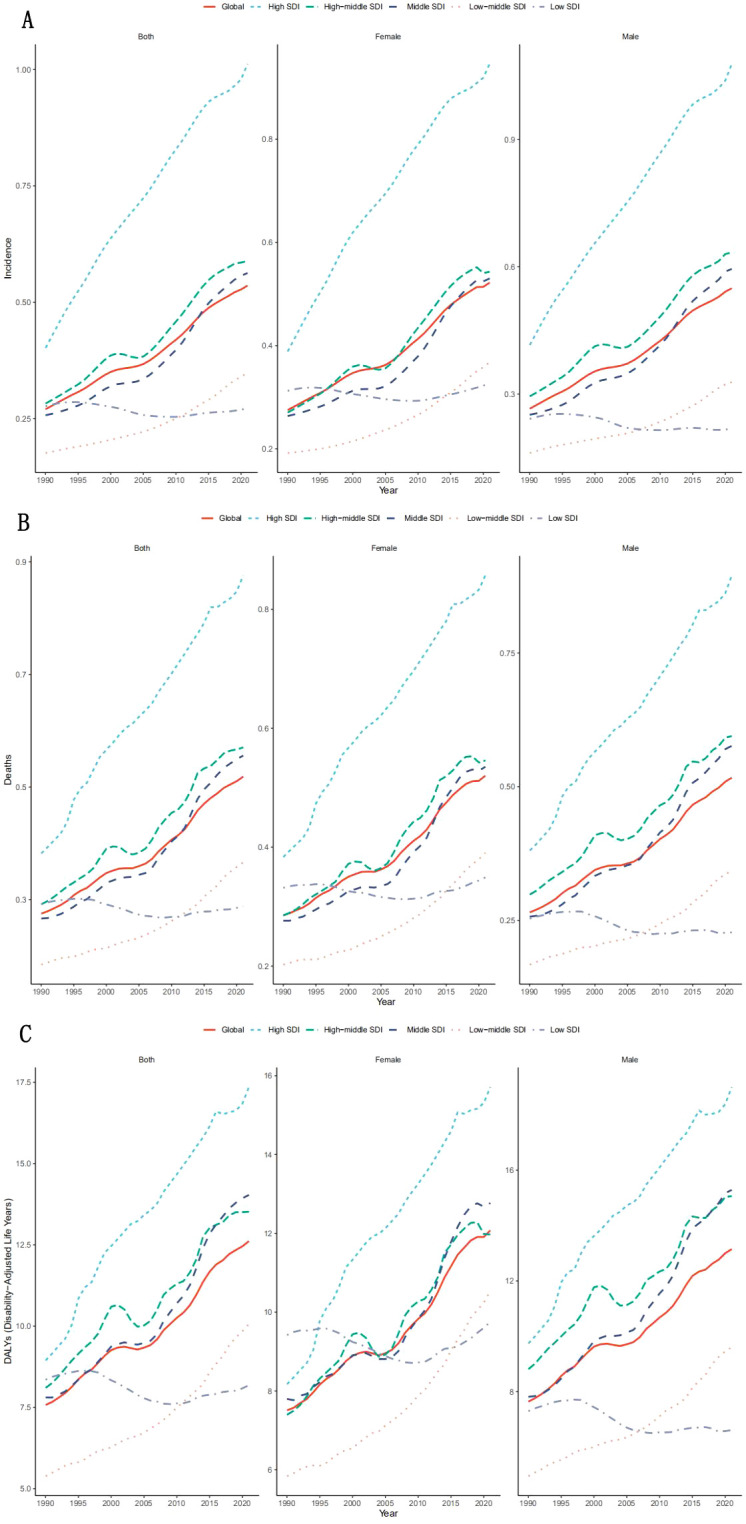
Trends in incidence **(A)**, mortality **(B)** and DALYs **(C)** for the disease globally and in the five SDI regions, 1990 to 2021.

The global incidence of NASH-related liver cancer (**Figure 5**) continued to increase from 0.3% (95%UI:0.2-0.3) in 1990 to 0.5% (95%UI:0.4-0.6) in 2021, an increase of approximately 98.5%. For example, the incidence rate of men in High SDI areas increased from 0.4% (95%UI:0.3-0.5) in 1990 to 1.1% (95%UI:0.9-1.4) in 2021, an increase of 160%, which is significantly higher than that in other regions. High SDI region: highest incidence and fastest growth, 1.0% in 2021 (95%UI:0.8-1.3), Low SDI region: lowest incidence but less fluctuating, 0.3% in 2021 (95%UI:0.2-0.4). Middle SDI and High, Middle SDI regions: Showing moderate growth, Middle SDI region in 2021 is 0.6% (95%UI:0.4-0.7). The incidence was higher in men than in women in all regions, with the largest difference in High SDI regions. It may be related to a higher risk of metabolic disorders (such as insulin resistance) in men, alcohol synergies, or hormonal differences.

The global mortality rate for NASH-related liver cancer also increased from 1990 to 2021, from 0.3%(95%UI:0.2-0.3) in 1990 to 0.5% (95%UI:0.4-0.6) in 2021 in men and 0.3% (95%UI:0.2-0.4) in women Increased to 0.5% (95%UI:0.4-0.6). Areas with a high SDI have significantly higher mortality rates than areas with a low SDI, with a high SDI rate of 0.876% (95%UI:0.7-1.1) in 2021 compared to 0.3% (95%UI:0.2-1.1) in areas with a low SDI. Death rates are also generally higher for men than for women. In the future, as obesity rates rise, the burden of NASH-related liver cancer is likely to increase further, especially in areas with high SDI.

Globally, DALYs for NASH-related liver cancer differ significantly by region and gender. The highest DALYs rate was found in the areas with high SDI, with 19.0 for males (95% UI: 15.1-24.0) and 15.7 for females (95% UI: 12.5-19.4), for a total of 17.4 (95% UI: 13.9-21.7). In contrast, low SDI areas had the lowest DALYs rates, 6.61 (95% UI: 4.41-9.50) for men and 9.8 (95% UI: 7.0-13.2) for women, for a total of 8.2 (95% UI: 5.9-11.2). This result highlights the importance of strengthening the prevention, early diagnosis and treatment of NASH and liver cancer globally, especially in high to burden areas.

### 3.5 Global disease burden of hepatocellular carcinoma attributable to MASLD attributable to risk factors

**Figure 6:**
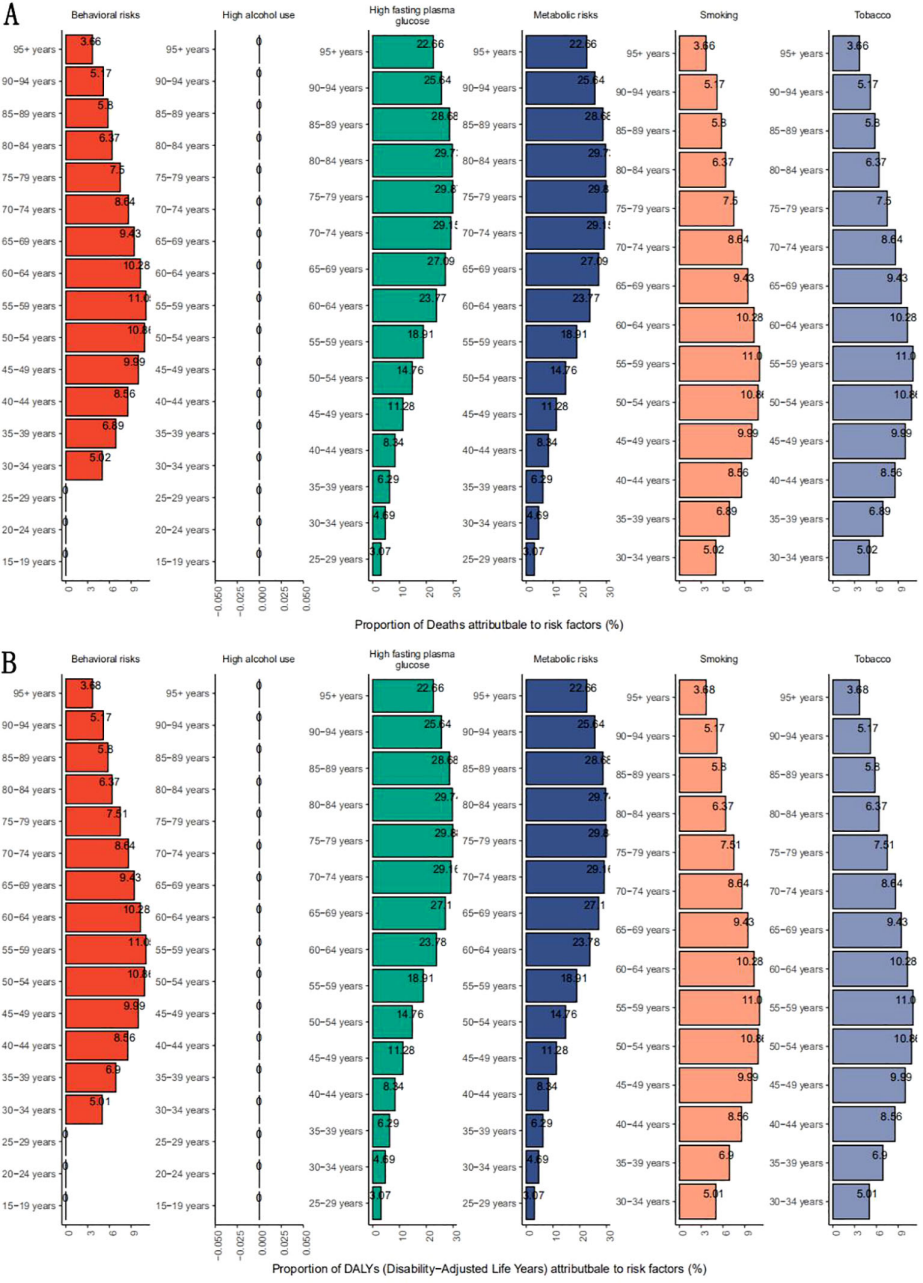
Percentage of mortality **(A)** and DALYs **(B)** rates due to disease by relevant risk factors in each age group.

Age and sex stratification revealed critical gradients, peaking at ages 85–89 years (6.31 per 100,000 men; 5.56 per 100,000 women) with narrowing gender gaps after age 75 (women ≥95 had 8.5% higher mortality than men).nonalcoholic fatty liver disease attributed to risk factors leads to GBD outcomes for liver cancer (**Figure 6**), and the main risk factors for death are metabolic risks (such as high fasting glucose) and behavioral risks (such as smoking), especially in older age groups. There was significant heterogeneity in the mortality of liver cancer due to metabolic risk of nonalcoholic fatty liver disease at all ages, with the metabolic risk (including high fasting glucose) reaching its peak in the 75–79 year age group at 29.87% (95% CI: 3.11-57.69%), the mortality rate increased significantly in the age group over 80 years, 29.73% (95% CI: 3.12-57.98%) in the 80–84 year group, and gradually decreased with age. (95+ year group: 22.66% (95%CI: 2.31-44.92%); The effects of smoking and tobacco use were significant in people aged 40–69 years, peaking at 11.05% (95% CI: 3.78-18.24%) in the 55–59 age group. The distribution of behavioral factors overlaps highly with that of smoking, suggesting that smoking is the dominant factor in behavioral risk.

### 3.6 Future projections of the global burden of disease for hepatocellular carcinoma attributable to MASLD

**Figure 7:**
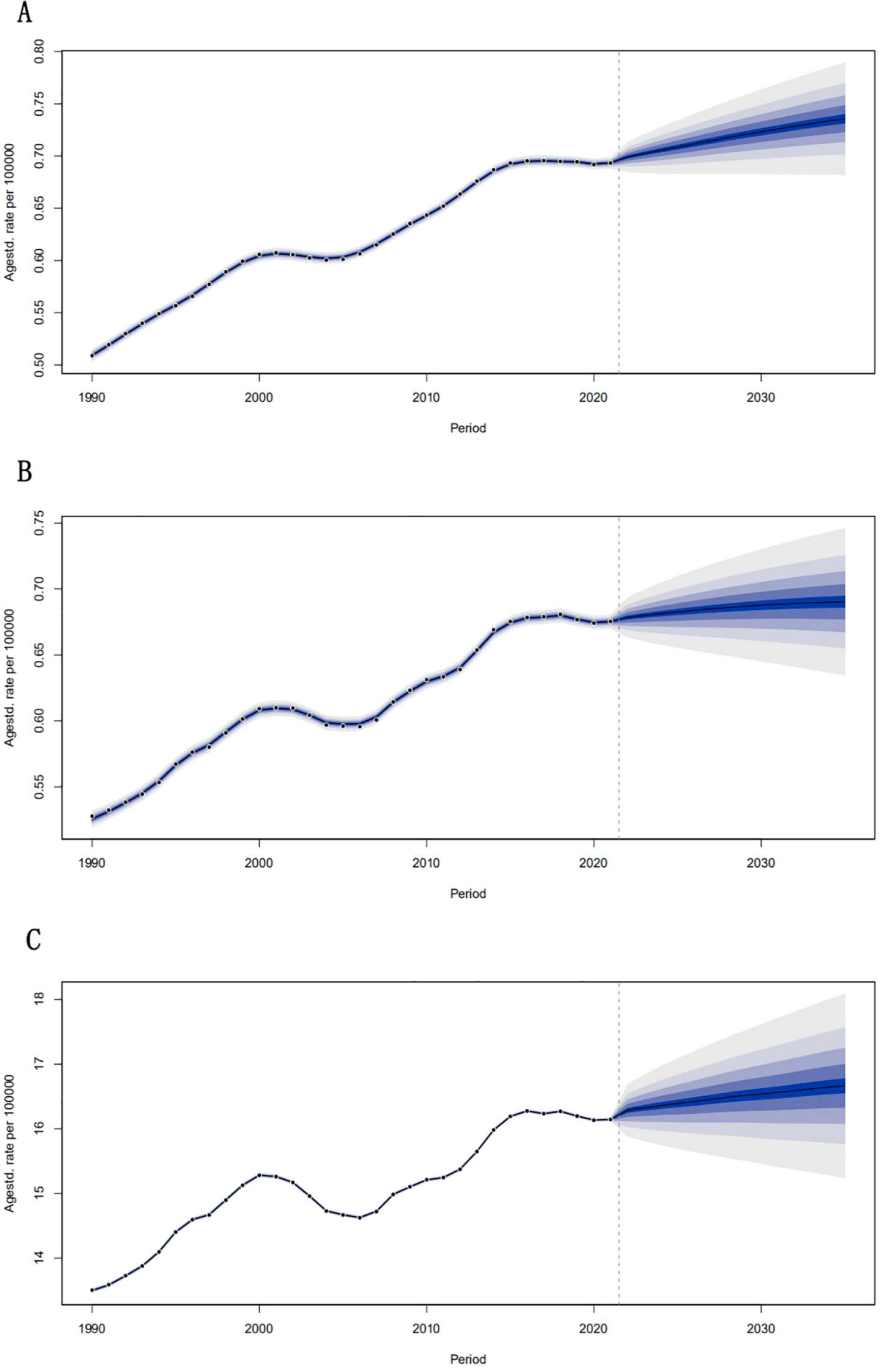
Global projections of incidence **(A)**, mortality, **(B)** and DALYs **(C)** for the disease from 2021 to 2035.

Projections indicated sustained growth, with ASIR rising 7% to 0.74 per 100,000 by 2035, primarily driven by aging (44% of DALY increase) and epidemiological changes (52% of incidence rise).To analyze post to 2021 trends in ASIR, ASMR, and ASDR for liver cancer development attributable to MASLD, we applied a Bayesian Age**-**Period**-**Cohort (BAPC) model to project these rates globally from 2021-2035 (**Figure 7**). The incidence of ASIR in all age groups of the disease will gradually increase globally. The analysis found that ASIR has steadily increased from 0.509 per 100,000 in 1990 to 0.693 per 100,000 in 2021, with an average annual growth rate of about 0.5%, and the disease burden of NASH-related liver cancer has increased year by year. A slight slowdown in the rate of ASIR growth between 2015 and 2020 (such as 0.692 per 100,000 in 2020) may be related to public health interventions or changes in diagnostic criteria. We then forecast that the global NASH-associated liver cancer ASIR will continue to rise from 2022 to 2035, reaching 0.736 per 100,000 in 2035. Its ASMR also increases year by year, from 0.526 per 100,000 in 1990 to 0.675 per 100,000 in 2021, with an average annual growth rate of about 0.5%. Then, we predict that ASMR will continue to rise from 2022-2035, reaching 0.690 per 100,000 in 2035, indicating that ASDR will further increase in the next 15 years. Finally, we analyzed the rise of ASDR from 13497 per 100,000 in 1990 to 16.143 per 100,000 in 2021, and predicted that ASDR would continue to rise from 2022-2035, reaching 16.663 per 100,000 in 2035.

### 3.7 Decomposition analysis of the global burden of disease in nonalcoholic fatty liver disease leading to hepatocellular carcinoma

**Figure 8:**
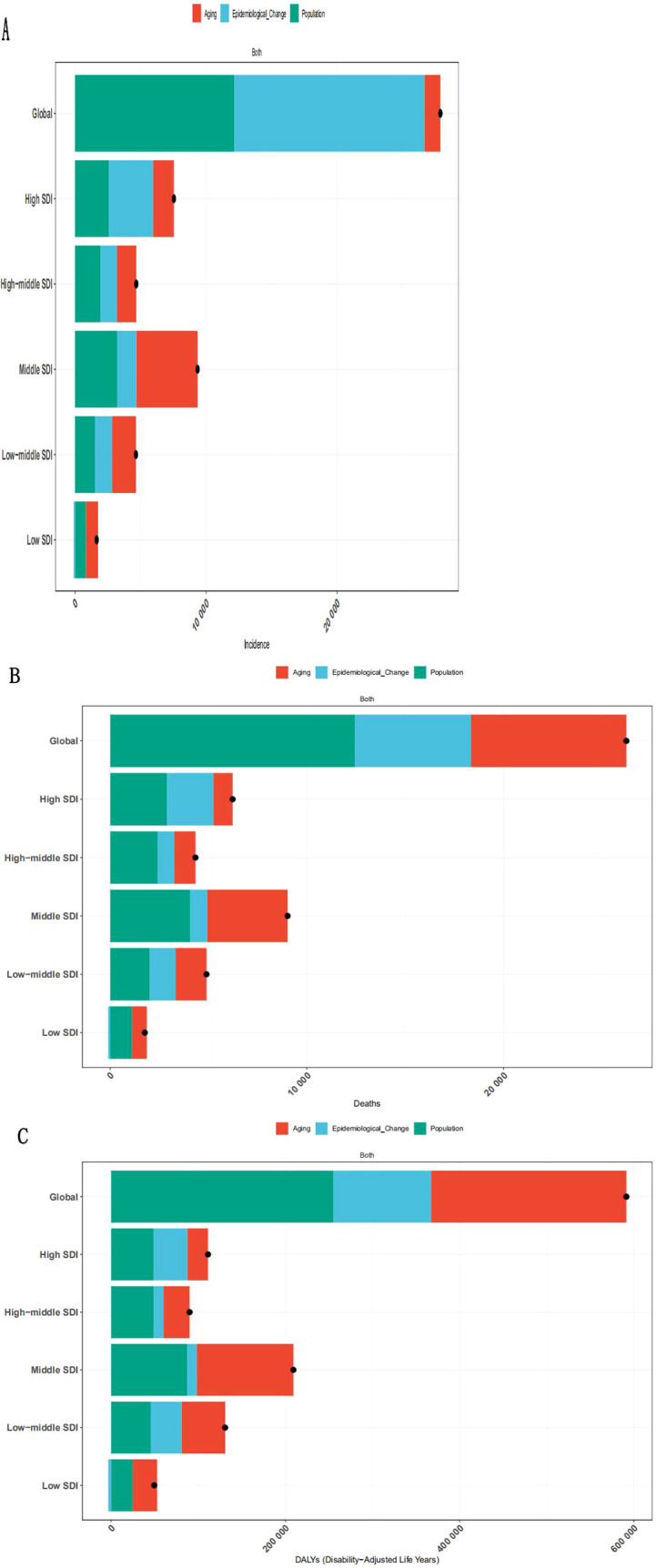
Schematic analysis of the decomposition of the incidence **(A)**, mortality **(B)** and DALYs **(C)** of the disease globally.

We performed a disaggregated analysis to assess trends in morbidity, mortality, and DALYs globally and in five SDI regions, focusing on the role of population growth, population aging, and epidemiological changes (**Figure 8**). The global incidence increased significantly to 27,877.45, indicating a higher incidence of NASH-related liver cancer worldwide. The overall difference can be broken down into aging (1216.06), population growth (12147.36), and epidemiological changes (14514.03). Epidemiological changes contributed the most to the overall difference, accounting for 52.06%; Among the 5 SDI regions, the increase of SDI was the highest, reaching 9351.14, in which epidemiological changes accounted for the most. Areas with high SDI are mainly affected by epidemiological changes (44.71%), while areas with low SDI are mainly affected by aging and population growth (55.94% and 50.04%, respectively). There are also significant differences in the mortality rate of the disease globally, with an overall global difference of 26,249.76, with population growth contributing the most, accounting for 47.38%. The drivers of disease burden varied across SDI regions. Population growth was the primary driver, accounting for 46.35% and 55.47% of the increase in high and high-middle SDI regions, respectively. In contrast, low SDI regions were predominantly influenced by a combination of population growth and population aging, which contributed 62.25% and 43.71%, respectively. The contribution of aging is significant in all SDI regions, especially in the middle and low SDI regions. The contribution of population growth is larger in the high SDI region, the middle and high SDI region and the low SDI region. The contribution of epidemiological changes is larger in the high SDI region and globally, but negative in the low SDI region (5.96%). We also analyzed the DALYs of the disease, with an overall difference of 591,461.98 on a global scale, indicating a high health burden of the disease, with population growth contributing the most. The burden drivers in different SDI regions are different, with high SDI regions and middle and high SDI regions mainly affected by population growth, while low SDI regions are mainly affected by aging and population growth.

## 4 Discussion

From 1990 to 2021, the global health burden of HCC associated with nonalcoholic steatohepatitis rose significantly, marked by increases in incidence, mortality, and DALYs. This trend is further evidenced by the positive EAPCs in morbidity, mortality, and DALYs, highlighting the accelerating worldwide impact of NASH-related HCC (27, 28). Notably, the incidence of NASH-associated HCC has risen dramatically, with a greater increase observed among men than women (29, 30). This pattern is especially evident in high-income regions such as North America, where lifestyle factors including obesity and metabolic syndrome are highly prevalent (31, 32). According to our findings, the global burden of NASH-related HCC increased by 25% between 1990 and 2021, exceeding the previous estimate of an 18% increase reported for 2010–2019 (33). This divergence can likely be explained by improved diagnostic protocols—such as the replacement of biopsy with non-invasive biomarkers in high-SDI regions—as well as the earlier adoption of screening in countries like Australia. In contrast, low- and middle-income areas, particularly those with low sociodemographic indices, demonstrate slower growth rates, which may be associated with underdiagnosis or limited healthcare infrastructure (34).

The disease burden of NASH-associated HCC varied considerably by region, with high-SDI areas showing markedly greater increases in mortality and DALYs. This pattern is largely attributed to the higher prevalence of obesity, metabolic syndrome, and Westernized diets in these regions, all of which are significant risk factors for the progression from NASH to HCC (35, 36). Lower-SDI regions, in comparison, exhibited more modest increases, likely reflecting competing health priorities and limitations in diagnostic infrastructure (37, 38). Nevertheless, the growing prevalence of metabolic risks, coupled with increasing SDI levels in these areas, signals a potential surge in NASH-related HCC in the coming years (30, 39, 40).

Despite their advanced healthcare systems, high-income countries face the counterintuitive challenge of a rising burden of NASH-related HCC. This trend is largely attributable to prevalent unhealthy lifestyles—characterized by high-calorie diets and sedentary behavior—which elevate the risk of obesity and metabolic disorders (41–43).However, in certain high-income regions such as Asia-Pacific, where proactive measures including early screening programs and stringent control of metabolic risk factors have been implemented, the burden of NASH-related HCC has stabilized or even declined (44).This contrast clearly demonstrates that the implementation of early prevention and intervention strategies is critical to mitigating the development and progression of NASH-related HCC (45).

Although gender differences in NASH-associated HCC converge at older ages, assertions regarding unique female risk factors require further validation. The observed elevation in mortality among women aged 95 and older may be attributable to a higher burden of comorbidities rather than NASH-specific biological mechanisms. Concurrently, the age-specific trend reveals that older populations, particularly those 75 years and above, bear the highest disease risk, with mortality rates rising exponentially with age (46). This pattern underscores both the long-term sequelae of NASH and the critical role of aging in exacerbating HCC risk. Moreover, while absolute mortality remains lower in younger age groups, a significant increase has been observed, indicating that NASH-associated HCC is emerging as a relevant health concern across all age demographics.

Metabolic risks dominated etiology, contributing 29.7% of deaths in ages 80–84, while behavioral risks such as smoking were prominent in younger cohorts, as evidenced by its contribution of 11.1% in the 55–59 age group. For example, smoking may accelerate the progression of NASH by exacerbating insulin resistance and oxidative stress (47); The combination of a high to sugar diet with sedentary behavior may further amplify metabolic disorders (48). In addition, regional differences suggest that metabolic risks dominate the burden of disease in countries with high SDI, while behavioral risks (such as increased smoking rates) in countries with low SDI may become new growth points in the future. For example, the proximity of mortality rates among women to men in the Caribbean may be associated with increased smoking rates and the prevalence of metabolic syndrome among women in the region. Compared with the GBD study in 2017, this study found that the attributional intensity of metabolic risk for the elderly increased significantly, suggesting that traditional metabolic interventions focusing on young and middle to aged people may not cover the elderly at high risk of MASLD to related liver cancer.

Future projections indicate that the burden of NASH-associated HCC will continue to rise, with incidence and mortality projected to increase steadily through 2035. This trend is driven by a global increase in obesity and metabolic syndrome, as well as an aging population. Regions with high SDI are expected to bear the heaviest burden, reflecting the challenges of managing lifestyle to related diseases despite advances in healthcare.

Decomposition analyses show that epidemiological changes, population growth, and aging are the main drivers of the global burden of NASH-related HCC (49). Decomposition analysis highlights key regional drivers: in high-SDI regions, 52% of the incidence growth was attributable to adverse epidemiological changes, exemplified by a surge in obesity rates. Conversely, low-SDI areas exhibited a negative epidemiological contribution (-5.96%), which may be explained by factors such as competing mortality from infectious diseases or persistent underdiagnosis. To address the heterogeneous burden patterns observed across regions, tailored public health interventions are imperative. In high-SDI settings, policy-driven measures such as sugar taxation and electronic health record-based MASLD screening should be prioritized to curb metabolic risk escalation. Conversely, in low-SDI regions, resource-appropriate strategies—including mobile ultrasound deployment coupled with community health worker training—are essential to overcome diagnostic barriers. For aging populations globally, integrating HCC surveillance into existing geriatric diabetes management programs offers a pragmatic approach to early detection. This stratified framework aligns with decomposition findings and targets region-specific drivers, including the contrast between epidemiological transitions in high-SDI regions and diagnostic gaps in low-SDI ones, to mitigate projected burden growth.

It is also worth mentioning that our analysis is subject to the inherent constraints of GBD data. Diagnostic heterogeneity across regions—ranging from biopsy-confirmed NASH in high-SDI areas to clinical diagnoses in low-resource settings—may bias burden estimates, particularly in regions with limited cancer registry coverage, notably sub-Saharan Africa. Underreporting of HCC deaths in regions dominated by competing mortality risks—for example, infectious diseases—further compounds uncertainty. This issue is compounded by the fact that the Bayesian age-period-cohort model projections rely on the assumption of constant future trends for metabolic risk factors, potentially failing to capture emergent public health crises or policy shifts. Additionally, our decomposition analysis quantified contributions from population aging and growth but could not fully disentangle interactions between coexisting risks such as diabetes and smoking. These limitations necessitate cautious interpretation of regional projections, especially in low-SDI contexts where data gaps are most pronounced.

## 5 Conclusion

The global burden of HCC attributable to nonalcoholic steatohepatitis increased substantially between 1990 and 2021, with significant disparities observed across sociodemographic index (SDI) regions. High-SDI regions—such as Australasia and North America—experienced accelerated growth in HCC incidence and mortality, with rates rising by 160%-200%, largely driven by obesity epidemics and aging populations. In contrast, low-SDI regions, including sub-Saharan Africa, exhibited elevated mortality despite lower incidence rates, highlighting critical gaps in diagnosis and treatment accessibility. Population aging alone accounted for 43.7% of the global increase in DALYs, underscoring its pivotal role as a key driver. Epidemiological projections indicate a further 7% rise in HCC incidence by 2035. To reverse this trajectory, priority interventions should include: implementing metabolic risk reduction strategies such as sugar taxes and enhanced diabetes control in high-SDI regions; integrating HCC surveillance into geriatric care systems to facilitate early detection; and deploying mobile diagnostic units to expand healthcare access in low-SDI settings.

## Data Availability

The original contributions presented in the study are included in the article/**Supplementary Material**. Further inquiries can be directed to the corresponding author.
